# Willingness-to-pay for a population-based-prostate-specific antigen screening for prostate cancer in Anambra State, Southeast, Nigeria: a contingent valuation study

**DOI:** 10.4314/ahs.v22i4.7

**Published:** 2022-12

**Authors:** Blessing Ifeoma Umeh, Brian Onyebuchi Ogbonna, Sunday Odunke Nduka, Jovita Ifeoma Nduka, Loveth Izuchukwu Ejie, Uchenna Adaobi Mosanya, Ikechukwu Obinna Ekwunife

**Affiliations:** 1 Department of Clinical Pharmacy and Pharmacy Management, Nnamdi Azikiwe University, Awka, Anambra State, Nigeria; 2 Department of Clinical Pharmacy and Pharmacy Management, University of Nigeria Nsukka, Enugu State Nigeria

**Keywords:** Willingness to pay (WTP), prostate-specific antigen (PSA), Population-based screening, prostate cancer, contingent valuation study, Anambra state, Nigeria

## Abstract

**Background:**

Early diagnosis of cancer precursors improves treatment outcomes. Organized screening for prostate cancer is still uncommon in Nigeria, and if it is added to the national health budget, it may necessitate additional co-financing alternatives.

**Objectives:**

The study aims to evaluate the maximum willingness- to- pay amount and acceptability of a Population-based screening for prostate cancer among a group of Nigerian men.

**Methods:**

The study was a cross-sectional survey-based study conducted among men drawn from different districts of the state. The payment card elicitation format was used to estimate the average maximum WTP amount. Multivariate Logistic regression was used to evaluate the correlates of WTP.

**Result:**

A total of 439(81.9%) participants were willing to pay for the screening while only 97(18.1%) of the participants rejected the screening. The average WTP amount was US$6.01(mean ± median ± SD 6.01±4.12±5.75). Residence and knowledge of the disease were the major predictors.

**Conclusion:**

The findings showed that men in Anambra state Nigerian were willing to pay an average of US$6.01 for the Population-based screening. Even though the stated WTP amount seems low compared to the conventional cost of opportunistic screening (between USD 21), the majority of the participants 439(81.9%) willing to pay for the screening should be capitalized upon in finding alternative financing options for the program.

## Introduction

Prostate cancer is said to be the second most common cancer in men and the fifth leading cause of cancer death worldwide. 1 In 2012, there were over 1.1 million estimated new cases of prostate cancer globally. 1 Blacks have the highest incidence of prostate cancer in the world 1. Nigeria has a hospital incidence of prostate cancer of 127/100,000 cases, with a prostate cancer risk of 2% in men older than 55years and a national prostate cancer pool of 110,000 cases. 2

Early detection is one of the major ways of detecting the precursors of cancer and enhancing treatment outcomes. Even though the mortality-reducing effect of Prostate-specific antigen (PSA) screening has not yet been sufficiently demonstrated, the PSA test might be a promising measure for prostate cancer screening. 3 In the United States, studies from the Surveillance Epidemiology and End Results database and the American Cancer Society (ACS) National Prostate Cancer Detection Project indicate that the widespread use of PSA testing has resulted in an obvious decline in the diagnosis of metastatic disease and more frequent diagnosis of localized cancers compared with historical controls. 4 Screening for prostate cancer is necessary to ensure early diagnosis and enhanced survival. Since increased PSA level is one of the markers for prostate cancer, it is worthwhile for elderly men to undergo these checks regularly. In developed countries like the USA where awareness about prostate cancer is high, the American Cancer Society recommends yearly digital rectal examination and PSA determinations for prostate cancer screening which is usually covered by health insurance. A Nigerian male population is an unscreened group. It has been shown that the incidence of Prostate cancer (PCA) is on the increase among Nigerian men just as in other men of African descent. 5 The cancer is also often aggressive and usually discovered at later stages. (6) In Nigeria, awareness is quite low 7 and screening for prostate cancer is usually opportunistic. Most cases are seen at the advanced stage of the disease, thus making survival difficult. Spending on late-stage cancer treatment adds to the financial burden of many families. 5 The annual mean income in Nigeria is US$1766 and most unskilled workers earn only US$440. 8 This implies that early detection through annual population-based screening for prostate cancer may be a more viable option for reducing the burden of the disease. Currently, there is no organized annual prostate screening program in Nigeria. Screening for prostate cancer is still not common. There is a need to explore options for the introduction of a mass screening program for prostate cancer in the country. For this to be feasible, it is necessary to ascertain the demand users place on such a program. This can be done by estimating their willingness to pay (WTP) values using the contingent valuation method. According to welfare economics, WTP can theoretically quantify how much value individual places on a health technology or an intervention. 8, 9,10,11,12 The value individuals place on a certain health intervention can guide policymaking in finding financing options like subsidies, co-payment, for implementation of such intervention.

To the best of our knowledge, no study has assessed the WTP of a Population-based PSA screening for prostate cancer program in Nigeria. This study, therefore, aimed to ascertain the WTP of a Population-based PSA screening for prostate cancer among a group of Nigerian men, the acceptance and factors influencing the amount they are willing to pay for such intervention.

## Materials and Methods

### Ethics approval

The research design and procedure were approved by the local ethics committee of the Nnamdi Azikiwe University Teaching Hospital Anambra State Nigeria. The anonymity and confidentiality of the participants were respected by the researchers.

### Study Design

This study, a questionnaire-based cross-sectional study was conducted in Anambra State, Nigeria, from April 2019 to October 2019. Anambra state was selected due to its accessibility and proximity to the researchers. The state is located at latitude 6.20°N and longitude 7.00°E with a total area of 4844 km. The inhabitants of Anambra State are mainly of Igbo ethnic nationality with a small population of Igala ethnic group. There are 21 local government areas (LGA) in the state. Anambra had by 2016 an estimated population of 5.5 million with a 2.8million male population 13. With an estimated 2.84% growth rate, the population of Anambra is expected to be about 6.358 million in 2021. 13. The citizens are predominantly Christians. There are three main religious denominations in the state; catholic, Anglican, and Pentecostal. A multistage random sampling technique was used. The first stage was the selection of two local government areas from each strata using simple random sampling of balloting after classifying the LGAs into urban, semi-urban, and rural areas. This was to ensure the inclusion of individuals from high, middle, and low socioeconomic classes in the study. Then the respondents were drawn from the three major religious groups in each of the selected local government areas using a proportionate allocation ratio where 50 percent of the participants were assigned to the Catholic Churches, 30% to Anglican Churches, and 20% to Pentecostal Churches. The basis for selecting the respondents using the various Churches as sampling was to frame the higher possibility of getting a large crowd within a short time.

The participants were informed during church service about the study. Those that gave their consent for the study after each church service were given the questionnaire to fill. Eligibility for the study included male participants who were between 20 and 75 years of age at the time of the data collection and who gave their consent for the study.

### Sample size determination

Using Anambra state with an estimated male population size of approximately 2.8 million confidence levels of 95 % and margin of error of 5%, 385 respondents (approximately 600 respondents were recruited to account for unusable questionnaires) were determined to be appropriate for the survey 14.

### Study Tool Validation

The adapted questionnaire was face validated using three senior faculty members to assess the presentation as well as the relevance of the questionnaire, and pilot tested with thirty participants that were excluded from the final study, to assess feasibility. Modifications were made to the questionnaire based on any identified problem(s). We hypothesize that if the mass screening program is a highly valued program among the study participants then it is most likely that trading will occur and our WTP result will be valid if: (i) the WTP amount shows a positive relationship with income i.e. those in higher economic strata been more willing to pay for the intervention; (ii) the screening rejecters (those participants that stated that if the screening was not free of charge they will not be willing to be screened) states zero or no WTP amount. The basis for the above assumptions is that; the use of payment card elicitation procedures in a contingent valuation study can mimic conventional purchasing behaviour. 8 The self-administered questionnaire was adapted from two related studies. 9, 10 The questionnaire was reviewed to suit the study's purposes. The final questionnaire consisted of three sections. Section A; contained general information and also assessed the socio-demographic characteristics of the respondents such as age, monthly income, marital status, etc. Section B; assessed respondents' knowledge of prostate cancer. Section C; contained a brief scenario description and the payment card used to assess the respondents' WTP for the screening.

### Willingness- to- pay Assessment

A contingent valuation approach using the payment card elicitation format with an open-ended question was used to estimate the average maximum willingness-to-pay (WTP) among the survey participants. The payment card elicitation format was chosen because it is more efficient for obtaining more information from participants. With the payment card, the participants will just be required to choose from the list of offered WTP amounts rather than being asked to provide their WTP amount which could result to non-response. Acceptance of the screening was assessed based on the response to the question; ‘What if the population-based screening is not free and you are supposed to pay out of pocket to get screened, will you be willing to pay for the screening? Respondents that gave positive responses were instructed with a follow-up question to indicate on the payment card with a tick mark symbol the maximum amount they were willing to pay, and cross mark amount they will never pay for the screening and state their reason for choosing the stated WTP amount. The presented prices on the scale ranged from 0 Naira to more than 12500 Naira (equivalent to US$0- US$34.34). The maximum amount on the payment card represents the usual cost of conventional PSA screening offered in most health facilities in Nigeria. The payment card has three different price ranges in incremental order and also contained an open-ended question where the respondents were required to state their WTP amount if their WTP amount was not represented on the scale. The maximum amount they were willing to pay was considered as their perceived monetary benefit of the intervention. This is in line with the welfare economics theory which states that the benefits individuals place on intervention is defined by their maximum willingness to pay for the intervention. The different payment card scales with a varied range of prices were randomly given to the respondents to avoid range bias. The prices on the payment card were written in Nigerian currency but the presented results are expressed in US dollars (NGN 364≈ US $ 1.00). (CBN, 2019).

### Data Analysis

The collected data were entered into Microsoft Excel (Microsoft Corporation, Redmond, Washington) for sorting and were checked for accuracy. They were then transferred into IBM Statistical Package for Social Sciences (IBM SPSS) for descriptive statistics, internal consistency, and logistic regression analyses. The WTP values (as mean [SD] with 95% CI) were calculated directly from the collected data. Responses to the WTP question were used as the dependent variables in the multivariate binary logistic regression. The responses were grouped into two: ‘screening acceptors; and ‘screening rejecters. Screening acceptance or rejection was measured based on the responses to the following question on the questionnaire: ‘Assuming you have to pay to get a PSA screening for prostate cancer in your community once every year, will you be willing to be screened’? A follow-up question was used to assess willingness to pay (WTP) amount. The participants who answered “NO” or gave a zero WTP on the payment card were classified as “screening rejecters”, while the ones who answered “yes” and indicated a positive value in the payment card were classified as “screening acceptors”. For the demand curve; at each given price on the scale, we determined the cumulative percentage of participants that were willing to pay for the program at that price plus all those who gave a higher WTP (i.e., tolerant individuals). 8 The value was plotted on a graph as the y-axis against price or WTP. 8 A multivariate binary logistic regression analysis using WTP values (positive WTP response and-zero WTP response) as the dependent variable and some socio-demographic factors as explanatory or independent variables were used to investigate the various factors influencing the WTP amounts.

## Result

### Characteristics of the respondents and awareness of the prostate disease

From the socio-demographic characteristics shown in Table 1; more than half of the respondents [355(66.2%)] were above 50 years of age, while 69(12.9%) respondents reside in rural communities. A higher proportion of the respondents [406(77%)] were married and 167(32.1%) reported monthly income of between US$ 274- 687.

**Table 1:** Socio-demographics of the respondents (n=536)

Variable	Frequency (n=536)	Percent (%)
Age(years)		
20–30	10	1.9
31–40	39	7.3
41–50	132	24.6
Above 50	355	66.2
Place of Residence		
Rural	69	12.9
Semi-urban	233	43.5
Urban	234	43.7
Denomination of the participants		
Catholic	262	48.9
Anglican	152	28.4
Pentecostal	122	22.7
Marital Status		
Married	406	77
Single	36	6.8
Divorced	42	8.0
Widowed	41	7.8
Co-habiting	2	4.0
Education		
No formal education	40	7.5
Primary	86	16.1
Secondary	129	24.2
Tertiary	163	30.5
Post tertiary	116	21.7
Occupation		
No Job	11	2.1
Farming	27	5.0
Self-employed	135	25.2
Civil servant	156	29.2
Business	146	27.3
Retiree	60	11.2
Monthly Income		
US$ <137 (<₦50,000)	136	26.2
US$ 137–274 (₦50,000–100,000)	150	28.8
US$ 274–687(₦100,000–250,000)	167	32.1
US$ 687–1373(₦250,000–500,000)	60	11.5
Above US$ 1373(₦500,000)	7	1.3

1US$=364 Nigerian Naira (₦)

Table 2 shows the respondent's awareness of prostate disease. Only a few of the respondents [39(7.4%)] have had a history of abnormal PSA results. Most of the respondents [313(60.4%)] have heard of prostate cancer while less than half of the respondents [142(27.6%)] knew about other diseases of the prostate. Health professionals were reported as the main source of their information on prostate diseases.

**Table 2:** Awareness of Prostate diseases (n=536)

Variable	Frequency (n=536)	Percentage (%)
**Diagnosed with Prostate disease**	58	10.8
**History of abnormal PSA**	39	7.4
**Ever heard of prostate disease**	142	27.6
**Ever heard of Prostate cancer**	313	60.4
**Source of Prostate disease awareness**		
Health professionals	87	42.9
Family or Friends	47	23.2
Newspapers or magazines	23	11.3
Television	20	9.9
Internet	18	8.9
Cannot remember	**8**	3.9

**NB**: the total number for some variables is not equal to the sample size because of missing data

### Average Willingness to pay (WTP) for the mass PSA screening for prostate cancer program and its predictors

**Figure 1:**
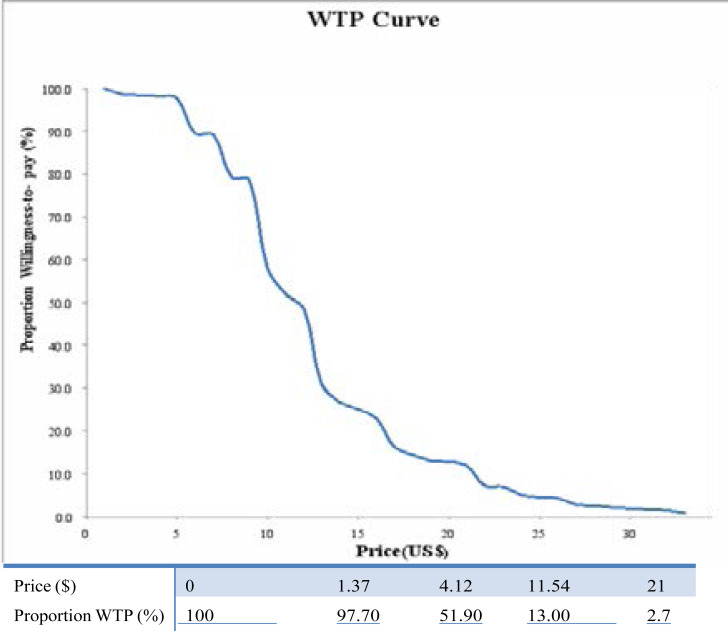
Relationship between Presented Price (US$) and Cumulative demand.

Table 3 shows the mean WTP and average amount that would never be paid by the participants. Most of the respondents 439(81.9%) provided a WTP value, only 97(18.1%) of the participants gave a ‘no’ or a zero response. The mean WTP amount stated by the respondents was US$6.01(Mean ±SD 6.01±5.73) while the most stated WTP amount was US$2.74. Most of the respondents 389(72.6%) stated US$12.69 as the amount they will not pay for the program. Figure 1 is the WTP demand curve, which represents the relationship between the hypothetical price of the mass screening program and the proportion of respondents agreeing to pay at each price level to get screened annually for prostate cancer. The shape and the negative slope of the demand curve are in line with the law of demand; that as the price of the screening increases the percentage of respondents willing to pay decreases.

**Table 3:** Average WTP, Amount not to pay for mass PSA screening and its predictors n=536

Statistics	WTP Per Screening (US$) n=536
Mean	6.01
Median	4.12
Mode	2.74
Percentiles	
20	1.92
90	13.93

**Amount not To Pay Per Screening (US$) n=389**	

Mean	**12.69**
Median	7.69
Mode	6.87
Percentile	
20	4.12
90	34.34

Dependent: Zero WTP amount (=0), n=97, Positive WTP amount (=1), n=439)						
Logistic regression	b	S.E	Sig.	b(exp)	95%CI lower	Upper
Variables						
Residence-Semi urban	-1.568	.487	.001	.209	.80	.541
Ever heard of prostate disease	.757	.342	.027	2.131	1.089	4.170
Heard of prostate cancer	.676	.327	.039	1.966	1.035	3.736
Model Chi-square	90.692					
Cox & Snell R square	0.170					
Nagelkerke R2	.275					

**NB:** Only significant independent variables (sociodemographic characteristics) shown

Multivariate logistic regression was conducted to determine if the combination of the selected variables could predict the likelihood of positive WTP for the screening among the respondents. The results of the logistics regression were statistically significant, X2(21) = 90.692, p=.0.000.

The result of the logistic regression shows that 97 (18.1%) of the respondents rejected the program (Table 3). Respondents who have heard about prostate diseases are more likely to accept the screening while respondents who reside in semi-urban areas were more likely to reject the screening. The predictive capacity of the model was between 17% to 27.5%. The b- coefficients from Table 3 shows that for a unit increase in knowledge of prostate diseases and or knowledge of prostate cancer there will be a probability of 2.131 and 1.966 increase in positive WTP amount respectively. Also, for a unit increase in the number of semi-urban dwellers, there will be a 0.209 decrease in the probability of positive WTP amount.

## Discussion

The study aimed to evaluate how much Nigerian men are willing to pay for a population-based PSA screening for prostate cancer. The study successfully established the average WTP amount among the study participants. Participants who are aware of the disease were more likely to pay for the screening, while those that reside in semi-urban (middle income) areas were more likely to reject the screening. This is in line with a similar study in Japan which also showed that income was a significant predictor of WTP for a mass prostate cancer screening. 15 Since awareness of the disease was the major predicting factor for positive WTP amount, more emphasis should be placed on creating avenues for the education of the populace on prostate cancer.

The study will have a practical impact on the implementation of annual prostate cancer screening for men. From the study, the acceptance of the screening was quite high; most of the respondents were willing to accept the annual screening. This is comparable to findings from a similar study which reported that men place more value on peace of mind and reassurance by confirming no sign of cancer than the long-term effects of such a program. 15

The high acceptance of the screening program indicates the likelihood of a successful implementation of an annual prostate cancer screening program in Nigeria. Co-financing options especially for people in semi-urban and rural locations of the state can be sought in other to enhance the uptake of the screening. A lot of factors like the inability to place value on a hypothetical good or service or seeing some intervention as one that should be provided by the government may have influenced the choice of the response of a few numbers of participants that rejected the intervention. Even though more than half of the participants were aware of prostate cancer, only a few of the participants knew about other diseases of the prostate. This shows the need for enhanced education and awareness of prostate health among the populace. Since health professionals were the most reported source of prostate cancer information by the participants, they should be empowered to intensify their efforts towards the provision of prostate cancer education to their patients. The high demand for screening is an indication that there is a need for the possible inclusion of such intervention in the national health budget. Increasing incidence of prostate cancer and late presentation of the cases should be of great concern to health policymakers and other funding agents like the national health insurance scheme in implementing a feasible means of creating awareness about the disease and increasing uptake of routine screening among men.

WTP usually indicates the demand individuals place on an intervention. 8, 15 It can serve as a strategy for finding financing options for a particular intervention. This may not be wholly applicable to developing countries because of other competing needs and a normally constrained budget. Mobilizing resources to pay for health care probably will mean a lot of financial consequences for the household. 8 Our study shows that participants with higher knowledge of the disease gave a more positive WTP amount and vice versa, this is in line with a lot of published WTP studies. 16, 17, 18, 19

With an estimated health expenditure of US$115 per capita and other competing health needs in Nigeria 19, the provision of free annual screening in Nigeria may not be a feasible strategy at the moment. It is necessary to consider other feasible means of supporting such intervention financially. The co-payment option for such intervention may be considered, the only hindrance to co-payment is that it may skew the screening uptake to only men in middle to higher income strata who are not up to half of the Nigerian population. This is evidenced by the fact that more than 58% of the Nigerian population lives with less than US$ 1.25 per day. 19 To ensure total inclusion of the whole populace in the intervention, subsidies, or provision of the intervention free-of-charge should be considered for those in the lower economic class especially the rural dwellers. For Construct validity, a simple theoretical proposition in economics is that demand for goods and services usually shows positive price elasticity. The higher the income, the more the WTP amount, this is consistent with our findings (Table 3).

The WTP amount obtained from our study should be considered in the light of bias associated with the payment card elicitation method of a contingent valuation study. The responses in the payment card are usually affected by presented prices (range bias). 20 To mitigate this bias, our study presented several versions of payment scales with a broad range of prices and also varied these versions randomly among the participants. There is also a tendency for the participants to state too low WTP amount (strategic bias) to influence the final price of the intervention or if they feel the intervention should be paid by the government. 8 ,20, 21 The small proportion of screening rejecters may have induced a strategic bias in our regression model and might have overestimated the odds ratio. 19, 20

The study was church-based, even though the state is a religious state small percentage of men who do not attend church may have been missed and the study included a few participants that have had the conventional PSA screening, these could have affected the stated WTP amount. To the best of our knowledge, this is the first study that assessed the WTP for mass PSA-based screening for prostate cancer in Nigeria. The timing of the study when the prevalence of the disease is high makes it more valuable in planning of viable strategy for prostate cancer control in Nigeria. The study was conducted in only one state in Nigeria, so the stated WTP amount may not be a full representation of the WTP for a mass PSA-based screening for prostate cancer by Nigerian men.

## Recommendation

Further studies should focus on other economic evaluations like; the cost of the program in other to conduct a full cost-benefit analysis (CBA).

## Conclusion

The findings showed that Nigerian men were willing to pay an average of US$6.0 for a mass PSA-based screening program for prostate cancer. Even though this amount falls short of the cost of conventional PSA- based screening offered in some hospitals in Nigeria, the high demand (81.9%) for this program should be capitalized upon in finding alternative financing options to make these services available to the populace. Co-payment options from both the populace and government could be a feasible mechanism for sustaining such intervention.

## Data Availability

The datasets for this study are available from the corresponding author on reasonable request.

## Abbreviations

WTP: Willingness to pay
NGN: Nigerian Naira
SD: Standard deviation
PSA: Prostate Specific Antigen
ACS: American Cancer Society
PCA: Prostate cancer
NPC: National Population Commission
CBN: Central Bank of Nigeria
CI: Confidence intervals
CBA: Cost-Benefit Analysis
UNDP: United Nation Development Programme

## Authors' information

IBU, IJN, and ILE are postgraduate students (Ph.D.) and lecturers in the Department of Clinical Pharmacy and Pharmacy Management, Nnamdi Azikiwe University Awka, Anambra State Nigeria. AUM is a postgraduate student (Ph.D.) and lecturer in the Department of Clinical Pharmacy and Pharmacy Management, University of Nigeria Nsukka, Enugu State, Nigeria. SON, OIE, and OBE are senior lecturers in the Department of Clinical Pharmacy and Pharmacy Management, Nnamdi Azikiwe University in Nigeria. OIE is also the coordinator of the Evidence-based health care research group, Nnamdi Azikiwe University Awka, Anambra State Nigeria.
